# Meningitis with septic shock resulting from odontogenic infection misdiagnosed as closed-lock in temporomandibular disorder: A case report and literature review

**DOI:** 10.1097/MD.0000000000034177

**Published:** 2023-07-07

**Authors:** Kunio Yoshizawa, Mitsuto Hanihara, Daiki Harada, Natsuhiko Myose, Hiroki Sakata, Takeshi Moriguchi, Akinori Moroi, Koichiro Ueki

**Affiliations:** a Department of Oral and Maxillofacial Surgery, Interdisciplinary Graduate School of Medicine, University of Yamanashi, Chuo City, Yamanashi, Japan; b Departments of Neurosurgery, University of Yamanashi, Chuo City, Yamanashi, Japan; c Department of Emergency and Critical Care Medicine, School of Medicine, University of Yamanashi, Chuo City, Yamanashi, Japan.

**Keywords:** meningitis, odontogenic infection, sepsis, temporomandibular disorder, trismus

## Abstract

Infections that spread to the pterygomandibular muscle can be misdiagnosed as temporomandibular disorder (TMD) because of the resulting difficulty in opening the mouth. Importantly, infection of the pterygomandibular space can extend to the skull base in the early stages, and a delay in therapeutic intervention can lead to severe complications.

**Patient concerns:**

A 77-year-old Japanese man with trismus after pulpectomy was referred to our department. This case report describes a rare instance of meningitis with septic shock caused by an odontogenic infection, initially misdiagnosed as TMD due to similar symptoms, leading to life-threatening complications.

**Diagnosis:**

The patient was diagnosed with sepsis and meningitis resulting from cellulitis in the pterygomandibular space caused by iatrogenic infection after pulpectomy of the right upper second molar.

**Interventions:**

After emergency hospitalization, the patient developed septic shock and required blood purification. Subsequently, abscess drainage and extraction of the causative tooth were performed. However, the patient developed hydrocephalus secondary to meningitis and underwent ventriculoperitoneal shunting to alleviate the condition.

**Outcomes:**

The infection was controlled and the patient level of consciousness improved following treatment for hydrocephalus. The patient was transferred to a hospital for rehabilitation on the 106th day of hospitalization.

**Lessons:**

Infections of the pterygomandibular space may be misdiagnosed as TMD, owing to the main symptoms of restricted mouth opening and pain on mouth opening. A prompt and appropriate diagnosis is crucial because these infections can lead to life-threatening complications. A detailed interview, along with additional blood tests and computed tomography (CT) scans, can aid in making an accurate diagnosis.

## 1. Introduction

Restricted mouth opening is a primary symptom of temporomandibular disorder (TMD). However, it can also occur as a result of tumors or infections invading the pterygomandibular space. Trismus is the most pertinent sign of pterygomoid muscle infection.^[1]^ Delayed diagnosis of severe acute infections can lead to irreversible and fatal complications. If the infection spreads to the pterygoid muscle, it can be misdiagnosed as TMD due to pain on mouth opening and limited mouth opening, which are the most common signs of TMD.^[2]^ Anatomically, the ascending development of infected foci in the pterygoid venous plexus can cause cavernous sinusitis and fatal meningitis. Therefore, clinicians must conduct thorough interviews, blood tests, and X-rays to diagnose patients and to differentiate between serious infectious diseases that mimic TMD.

Bacterial meningitis is a serious and often fatal disease, with a mortality rate of 20%.^[3]^ Prognostic factors for bacterial meningitis, which can be assessed as early as 1 hour after admission, have been identified,^[4]^ including age, heart rate >120 beats/min, Glasgow Coma Scale (GCS), cranial nerve palsies, cerebrospinal fluid (CSF) cell count <1000/mm^3^, and detection of gram-positive cocci in CSF. The incidence of meningitis sequelae is high, with reported rates of 31%, primarily hearing loss (21%) and hydrocephalus (7%).^[5]^

We present an extremely rare case of meningitis with septic shock resulting from an odontogenic infection after pulpectomy, which was initially misdiagnosed as TMD owing to overlapping symptoms, resulting in life-threatening complications.

## 2. Consent

Written informed consent was obtained from the patient legal guardian/next of kin for publication of the case and accompanying images.

## 3. Case report

**Figure 1.**
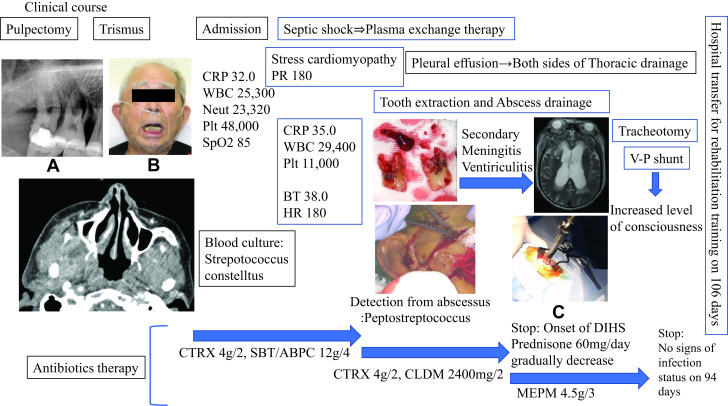
Laboratory and imaging results and procedures from the referral. (A) Dental radiographs from the initial visit show the vertical root fractures in the right upper first molar and the right upper second molar after the pulpectomy. (B) Facial photograph taken during emergency admission reveals inconspicuous swelling of the right side of the face, resulting in limited mouth opening. (C) The skull was opened during the ventricular drainage procedure.

A 77-year-old Japanese male presented to his private general dentist with chronic spontaneous pain in his right upper molars (Fig. 1: clinical course from the initial visit to transfer). The medical history included chronic obstructive pulmonary disease caused by long-term smoking. The dentist diagnosed the patient with pulpitis and performed pulpectomy (Fig. 1A). The dentist also noted a root fracture in the right upper first molar (Fig. 1A), which was treated with occlusal adjustment as an emergency measure. During the pulpectomy of the right upper second molar, the patient required wide mouth opening and experienced discomfort in the temporomandibular joint area. Three days after the procedure, the patient experienced sudden limited mouth opening and pain. In his family dentistry, he was diagnosed with acute TMD with closed-lock symptoms due to wide mouth opening during dental treatment and received loxoprofen sodium hydrate and mouth opening training. However, his symptoms worsened and he had difficulty eating. Ten days after the pulpectomy, he was urgently admitted to our department because of his poor general condition.

**Figure 2.**
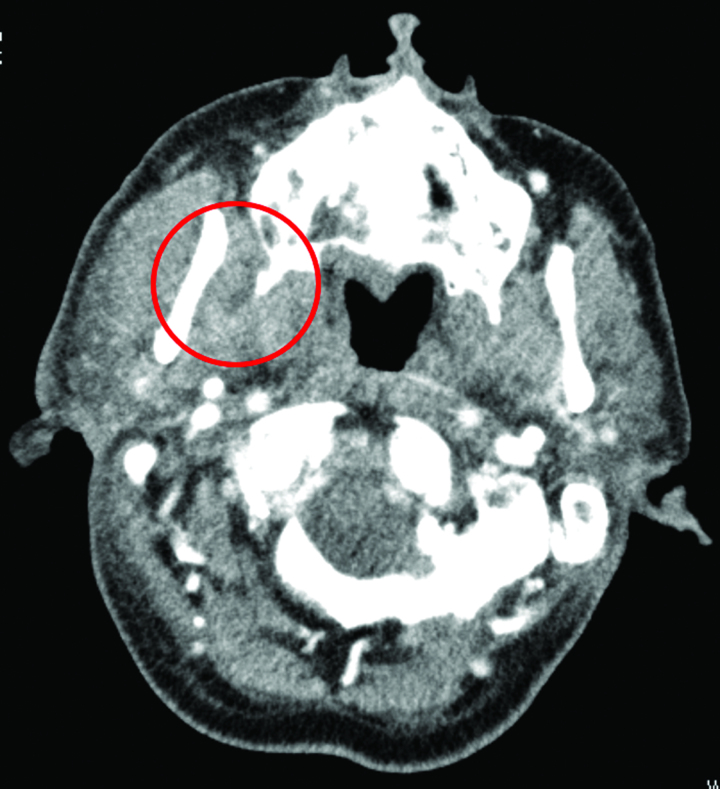
Contrast-enhanced CT findings on the first day of admission. An abscess formed between the mandibular branch and the pterygoid process. Buccal cortical bone resorption was observed at the root apex of the maxillary second molar, adjacent to the abscess. CT = computed tomography.

Upon examination, the patient exhibited no facial or oral swelling or redness but had a maximum mouth opening of 5 mm with pain on opening (Fig. 1B). He was conscious, but had difficulty walking. His body temperature and pulse rate were 36.7°C and approximately 90 beats/min, respectively, and his blood pressure was normal. The patient had a low SpO_2_ of 85; thus, oxygen was administered at 2 L/min via a nasal cannula. Despite the initial diagnosis of TMD, the patient medical interview suggested dental infection of the pterygomandibular space. Blood tests, blood cultures, and contrast computed tomography (CT) were performed (Fig. 2), and antimicrobial therapy with intravenous ceftriaxone sodium hydrate (4 g/day) and sulbactam/ampicillin (12 g/day) was initiated immediately given the results. Blood tests revealed a c-reactive protein (CRP) level of 32 mg/dL, White blood cell count of 25,300/μL, neutrophil count of 23,320/μL, and platelet count of 48,000/μL, and severe infection was diagnosed. Blood cultures were positive for facultative anaerobic *Streptococcus constellatus*, which is a member of the oral flora.

The patient septic condition continued to worsen, as evidenced by elevated infection markers in blood tests the day after emergency admission (CRP: 35 mg/dL; White blood cell count: 29,400/μL; platelet count: 11,000/μL; interleukin-6 [IL-6]: 18,119 pg/mL), as well as tachycardia (180 beats/min) and a decreased level of consciousness. As a result, the patient was transferred to the intensive care unit (ICU) with a diagnosis of sepsis. To address the septic condition, the patient was intubated and placed on a ventilator, and blood purification therapy was initiated upon admission to the ICU.

**Figure 3.**
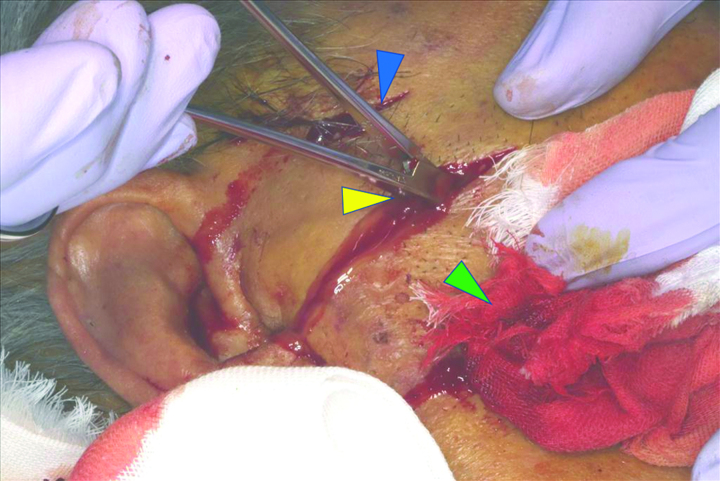
Abscess drainage using an extraoral incision approach. Blue arrow indicates the drainage puncture site in the inferior temporal space. Yellow arrow indicates the drainage punctures in the pterygomandibular space, which drained pale yellow pus. Green arrow indicates the drainage puncture site into the mandibular body.

**Figure 4.**
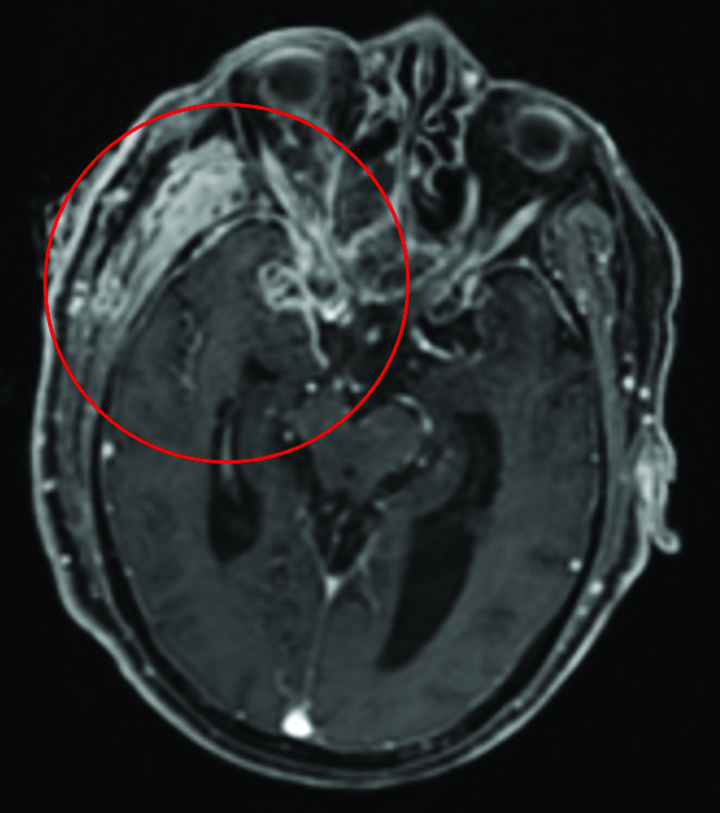
Skull opening during the ventricular drainage procedure.

The day after ICU admission (the third day of hospitalization), the patient underwent extraction of the causative tooth, the right maxillary molar, and drainage of the abscess in the pterygomandibular space through an extraoral incision (Fig. 3). *Peptostreptococcus*, an obligate anaerobe of the oral flora, was identified by culture testing of the drained abscess. After the drainage procedure, CRP and IL-6 gradually decreased to CRP: 1.6 mg/dL; IL-6: 14.1 pg/mL, and the patient respiratory status stabilized, prompting an attempt to extubate the ventilator. However, the patient level of consciousness remained unchanged, with a GCS score of eye opening of 1, best verbal response of 1, and best motor response of 3 (E1V1M3) on the 16th day of admission. Contrast-enhanced magnetic resonance imaging (MRI) revealed inflammation in the right optic nerve tract that continued into the skull, leading to a diagnosis of meningitis (Fig. 4). The patient CSF examination showed high cell counts (778 counts) and protein levels (122 mg/dL) with low sugar levels (32 mg/dL), indicating bacterial meningitis, although CSF culture tests were negative. Ceftriaxone with good spinal fluid transfer^[6]^ and clindamycin, which showed good drug sensitivity on the abscesses in this case, were chosen for treatment; however, meningitis persisted, and the MRI results on day 36 of hospitalization revealed hydrocephalus. Subsequently, the patient developed a pyothorax.

Unilateral thoracic drainage was performed on days 38 and 45 of hospitalization, resulting in improvement in the patient respiratory condition. On day 46 of hospitalization, tracheotomy, endoscopic third ventriculostomy, and ventricular drainage were performed because of the development of hydrocephalus. CT imaging on day 10 after ventricular shunting (admission day 56) revealed a reduction in ventricular diameter and edema of the periventricular White matter accompanied by improvements in the patient level of consciousness, grasping reflex, and spontaneous eye opening.

On the 63rd day of hospitalization, the patient developed drug-induced hypersensitivity syndrome, and ceftriaxone sodium hydrate and clindamycin phosphate were suspected drugs. Therefore, steroid medicine was administered, and antibiotic treatment was changed to meropenem, which was sensitive in the CSF culture test. When meningitis recurred during treatment, coagulase-negative *Staphylococcus* was detected in the CSF, which was sensitive to carbapenems and vancomycin. Therefore, meropenem was selected as the preferred antibiotic. Following the change in antibiotics, the truncal drug eruption caused by the drug-induced hypersensitivity syndrome gradually subsided.

After removal of the ventricular drainage on day 64 of hospitalization, the patient level of consciousness gradually decreased, and CT showed reenlargement of the ventricles. Therefore, a ventriculoperitoneal shunt was placed on the 88th day of hospitalization to address the recurrent hydrocephalus. After placement of the ventriculoperitoneal shunt, the patient level of consciousness improved and his GCS score increased to E4V1M3. Despite management with tracheostomy, the patient no longer required oxygenation and could begin rehabilitation. On day 106 of hospitalization, the patient was transferred to the hospital for rehabilitation.

## 4. Literature review

To find English publications, a literature search was conducted in PubMed on February 4, 2023, using the following search terms: meningitis ([MeSH Terms]) AND (Focal Infection, Dental [MeSH Terms]). In total, 7 English publications were found,^[7–13]^ reporting on 10 patients (7 males and 3 females with a mean age (± standard deviation) of 45.8 ± 17.0; Table 1) who developed meningitis after dental treatment. The present case is the oldest reported case and the only one that occurred after pulpectomy. In 9 of the 11 cases (including the present case), the causative tooth was in the maxilla; in the remaining 2 cases, the causative tooth was in the mandible. Similar to the present case, the most common causative tooth site was the maxillary molar region. The most common time of meningitis onset is 2 to 4 weeks after dental treatment, as in the present case. Previous studies have also found that meningitis often develops within 4 weeks of dental treatment.^[14]^

**Table 1:** Literature on meningitis caused by dental infections.

Author	Publication yr	Case no	Sex	Age	Site of dental infection	Treatment	Initial medical symptoms	Time to meningitis from dental treatment*
Hedstrom	1980	1	F	23	No details (in the upper jaw)	Deep caries treatment	Fever, chills, and headache	6 wk
		2	M	36	Three sites in the upper jaw (periapical destruction)	No details	Fever, headache, and tiredness	2 wk
		3	M	75	Bilateral upper lateral incisors (periapical destruction)	No details	Recurring fever with 4–5-d afebrile intervals	6 mo
		4	M	31	Left upper first premolar (apical periodontal cyst)	Dosing of *Penicillin*	Fever and headache	3 wk
Zachariades	1986	5	M	34	Impacted lower left third molar (pericoronitis)	Extraction	Trismus	2 mo
Fernando	1988	6	M	67	First and second incisors on left lower side (pulpitis)	Extraction	Severe headache, photophobia, neck stiffness, and fever	6 h
Montejo	1998	7	F	48	No details	Extraction	Severe fever and heavy headache	48 h
Colbert	2011	8	M	49	Left maxillary third molar (apical periodontitis)	Extraction	Ophthalmoplegia, ptosis, diplopia, frontal headache, and night sweats	2 wk
Hobson	2011	9	F	35	Left maxillary third molar (pericoronitis)	Extraction	Severe headache, facial swelling and mental status change	2 wk
Jose	2014	10	M	60	Right maxillary second molar (gingival inflammation)	Refusal of treatment	Periorbital headache and blurring of vision	4 wk
Present case	2022	11	M	77	Right maxillary second molar (pulpitis)	Pulpectomy	Trismus	2 wk

* includes cases where treatment was refused or only medical symptoms were observed with dental occult infections.

## 5. Discussion

The pterygomandibular space is located posterior to the maxilla between the mandibular ramus and pterygoid process of the sphenoid bone. It contains important structures such as pterygoid and pterygoid venous plexuses. Infections and abscesses in this area often cause trismus without obvious signs of inflammation such as redness or swelling of the mouth or face. In rare cases, infection and inflammation spreading along the venous plexus to the cranium can lead to potentially life-threatening complications, such as meningitis or brain abscesses. Therefore, before diagnosing TMD following the presentation of trismus, a thorough medical interview, blood tests, and CT should be performed to exclude restricted mouth opening due to infection. Although the initial treatment for TMD with trismus is typically conservative, it is important to rule out potentially life-threatening diagnoses such as severe infection before making a definitive diagnosis. In cases such as the present case, where an ascending dental infection presents with clinical symptoms similar to those of TMD, prompt blood tests and CT scans based on a detailed medical interview are crucial.

One potential lesson learned from the misdiagnosis in the present case was the importance of not prematurely assuming a diagnosis based solely on the patient reported symptoms. In the current case, the patient complaints of discomfort in the temporomandibular joint (TMJ) were attributed to mechanical stress from pulpectomy, leading to a misdiagnosis of acute closed locking of the TMJ. However, CT revealed that the true cause of the patient symptoms was an ascending dental infection that formed a perforation from the root apex of the maxillary second molar to the buccal cortical bone and spread to the lateral pterygoid muscle area around the TMJ.

Although several cases of meningitis after tooth extraction or uncontrolled infected teeth have been reported, meningitis after minimally invasive dental procedures, such as pulp extraction, has not been reported previously. Despite the distance between the maxillary molars and cerebral meninges, there are potential anatomical pathways for the spread of the infection beyond hematogenous dissemination. Venous drainage around the root apex of the maxillary molar connects to the pterygoid plexus, which, in turn, connects to the cavernous sinus and meninges. As illustrated in the present case, infection of the root apex of a maxillary tooth can potentially spread to the pterygoid plexus, which is connected to the cavernous sinus.^[15]^ It has been suggested that during yawning or mastication, reverse venous flow may occur from the pterygoid plexus to the maxillary vein because of the powerful contraction of the medial and lateral pterygoid muscles, in which the pterygoid plexus is enmeshed. Therefore, in the present case, it is possible that during pulpectomy, the bacteria causing pulpitis were pushed into the pterygoid plexus due to the mechanical stress of mouth opening and the thrust of the instrument. The MRI revealed inflammation and an abscess around the cavernous sinus and the adjacent optic nerve, indicating an ascending infection from the root apex of the maxillary molar that resulted in meningitis through the cavernous sinus.

When presenting with acute trismus, it is important to consider differential diagnoses such as TMD and tetanus infection; however, ruling out infection of the pterygomandibular space should be a primary concern. This requires thorough evaluation, including blood tests and CT scans, because the patient may exhibit few symptoms beyond trismus.

## 6. Conclusion

This case report highlights 2 important lessons. First, even minimally invasive dental procedures, such as pulpectomy, can lead to life-threatening meningitis in dental infections; therefore, dentists should carefully monitor any changes in trismus or the patient general condition after treatment. Second, an infection that spreads to the pterygoid plexus can present with symptoms similar to those of TMD. Appropriate diagnostic tests, such as blood tests and CT scans, should be performed promptly to avoid delays in treatment.

## Acknowledgments

We would like to thank Enago (www.enago.jp) for the English language review.

## Author contributions

**Conceptualization:** Koichiro Ueki.

**Data curation:** Mitsuto Hanihara, Takeshi Moriguchi.

**Investigation:** Mitsuto Hanihara.

**Resources:** Kunio Yoshizawa, Mitsuto Hanihara, Daiki Harada, Natsuhiko Myose, Hiroki Sakata, Akinori Moroi.

**Supervision:** Mitsuto Hanihara, Daiki Harada, Natsuhiko Myose, Hiroki Sakata, Takeshi Moriguchi, Akinori Moroi, Koichiro Ueki.

**Visualization:** Kunio Yoshizawa.

**Writing – original draft:** Kunio Yoshizawa.

**Writing – review & editing:** Kunio Yoshizawa, Mitsuto Hanihara, Daiki Harada, Natsuhiko Myose, Hiroki Sakata, Takeshi Moriguchi, Akinori Moroi, Koichiro Ueki.

## References

[1] Brown RS Johnson CD Fay RM. The misdiagnosis of temporomandibular disorders in lateral pharyngeal space infections--two case reports. Cranio. 1994;12:194–8. 7813033 10.1080/08869634.1994.11678019

[2] Ogi N Nagao T Toyama M. Chronic dental infections mimicking temporomandibular disorders. Aust Dent J. 2002;47:63–5. 12035960 10.1111/j.1834-7819.2002.tb00305.x

[3] Brouwer MC Tunkel AR van de Beek D. Epidemiology, diagnosis, and antimicrobial treatment of acute bacterial meningitis. Clin Microbiol Rev. 2010;23:467–92. 20610819 10.1128/CMR.00070-09 PMC2901656

[4] Weisfelt M van de Beek D Spanjaard L. A risk score for unfavorable outcome in adults with bacterial meningitis. Ann Neurol. 2008;63:90–7. 17823938 10.1002/ana.21216

[5] Jit M. The risk of sequelae due to pneumococcal meningitis in high-income countries: a systematic review and meta-analysis. J Infect. 2010;61:114–24. 20433866 10.1016/j.jinf.2010.04.008

[6] van de Beek D Brouwer MC Thwaites GE. Advances in treatment of bacterial meningitis. Lancet. 2012;380:1693–702. 23141618 10.1016/S0140-6736(12)61186-6

[7] Montejo M Aguirrebengoe K. Streptococcus oralis meningitis after dental manipulation. Oral Surg Oral Med Oral Pathol Oral Radiol Endod. 1998;2:126–7. 10.1016/s1079-2104(98)90413-9 9503443

[8] Fernando IN Phipps JS. Dangers of an uncomplicated tooth extraction. A case of Streptococcus sanguis meningitis. Br Dent J. 1988;165:220. 3224048 10.1038/sj.bdj.4806573

[9] Zachariades N Vairaktaris E Mezitis M. Cerebral abscess and meningitis complicated by residual mandibular ankylosis. A study of the routes that spread the infection. J Oral Med. 1986;41:14–20. 3457110

[10] Hedström SA Nord CE Ursing B. Chronic meningitis in patients with dental infections. Scand J Infect Dis. 1980;12:117–21. 7375823 10.3109/inf.1980.12.issue-2.08

[11] Colbert S Cameron M Williams J. Septic thrombosis of the cavernous sinus and dental infection. Br J Oral Maxillofac Surg. 2011;49:e25–6. 20691517 10.1016/j.bjoms.2010.07.004

[12] Hobson DTG Imudia AN Soto E. Pregnancy complicated by recurrent brain abscess after extraction of an infected tooth. Obstet Gynecol. 2011;118(2 Pt 2):467–70. 21768856 10.1097/AOG.0b013e31822468d6

[13] Jose A Nagori SA Bhutia O. Odontogenic infection and pachymeningitis of the cavernous sinus. Br J Oral Maxillofac Surg. 2014;52:e27–9. 24703382 10.1016/j.bjoms.2014.03.005

[14] Hollin SA Hayashi H Gross SW. Intracranial abscesses of odontogenic origin. Oral Surg Oral Med Oral Pathol. 1967;23:277–93. 5226436 10.1016/0030-4220(67)90138-7

[15] Ingham HR Kalbag RM Tharagonnet D. Abscesses of the frontal lobe of the brain secondary to covert dental sepsis. Lancet. 1978;2:497–9. 79867 10.1016/s0140-6736(78)92220-1

